# A Developmental Role for Fatty Acids in Eukaryotes

**DOI:** 10.1371/journal.pbio.0020293

**Published:** 2004-08-31

Health food stores have long hawked fish oil capsules as a cure-all for everything from migraines to heart disease. And though such claims are often weak on scientific evidence, fish oil, it turns out, is no snake oil. A recent review of scientific studies concludes that omega-3 fatty acids can indeed protect against heart disease, and the American Heart Association now recommends fish oil capsules for patients with coronary heart disease.

Fatty acids come in hundreds of varieties, distinguished primarily by their structure, which in turn determines their physiological role. Unlike proteins or genes—which are polymers made up of amino acids and nucleotides, respectively—fatty acids are a large group of compounds containing long chains of carbon and hydrogen atoms with a carboxylate group (acid) attached at the end. It is this asymmetrical chemical configuration that gives fatty acids their unique properties. Fatty acid diversity comes from variations in the length of the carbon chain and in the number of double bonds between carbons. Fatty acids with one or more double bonds are called unsaturated fatty acids.

Fatty acids play an essential role in metabolism, providing the cell with a concentrated source of energy, and form the structural foundation of the cell membrane, where they are most conspicuous and perhaps best understood. Long-chain (unbranched) fatty acids, which run ten to 22 carbons long, are the most common fatty acids in animal cells and the most studied. One much less understood class of fatty acids—the monomethyl branched-chain fatty acids (mmBCFAs)—has been found in organisms from bacteria to humans, but its role remains obscure. In this issue of *PLoS Biology*, Marina Kniazeva et al. explore the origin and function of mmBCFAs in the worm Caenorhabditis elegans and find that these relatively obscure fatty acids play a crucial role in growth and development.

mmBCFAs are abundant in diverse genera of bacteria, which use a supply of branched-chain amino acids and enzymes to assemble the fatty acid chains. mmBCFA biosynthesis has been characterized in bacteria, but not in eukaryotes. (Worms, and humans, are eukaryotes; our cells have nuclei.) Here, Kniazeva et al. identified worm genes that are homologous to the gene that codes for an enzyme called elongase in another eukaryote, yeast. Elongases are enzymes that extend the length of fatty acid chains by two carbons. To see what kind of fatty acid molecules the homologous worm genes were synthesizing, the authors used a technique called RNA interference (RNAi) to “silence” the genes' expression in the worms. Surprisingly, two of the eight inhibited genes had a specific effect on branched-chain fatty acid levels: *elo-5* and *elo-6*.

Inhibiting *elo-5* function had deleterious effects on the growth and development of the worms. The progeny of worms treated as embryos with RNAi for *elo-5* stopped growing at the first larval stage, while the progeny of worms treated at later stages developed to adulthood but got progressively sicker and showed reproductive problems. These defects were corrected when the researchers fed the mmBCFAs directly to the worms, indicating that these mmBCFAs are essential for normal larval growth and development.

Given the widespread distribution of mmBCFAs in organisms as diverse as bacteria and humans, it's perhaps not too surprising that they regulate essential physiological functions during animal development. It's still not clear, however, what all the components of the fatty acid manufacturing machinery are or how an organism monitors production levels. And though it's still an open question as to how these ubiquitous molecules function in mammals, the fact that they have been conserved throughout evolution underscores their importance—and suggests they may play a similar role.

